# Genetic diversity of *Plasmodium falciparum* among asymptomatic pregnant women on intermittent preventive treatment with sulfadoxine-pyrimethamine in Nigeria

**DOI:** 10.4314/ahs.v23i1.80

**Published:** 2023-03

**Authors:** Rita A Olowe, Johnson A Ojo, Roland I Funwei, Segun I Oyedeji, Olugbenga A Olowe, Bolaji N Thomas, Olusola Ojurongbe

**Affiliations:** 1 Ladoke Akintola University of Technology, Department of Medical Microbiology and Parasitology; 2 Babcock University, Department of Pharmacology; 3 Federal University Oye-Ekiti, Department of Animal & Environmental Biology; 4 Rochester Institute of Technology, Department of Biomedical Sciences

**Keywords:** Malaria, pregnancy, allelic frequency, multiplicity of infection, Merozoite surface protein, glutamate-rich protein

## Abstract

This study investigated the genetic diversity of *Plasmodium falciparum* among asymptomatic pregnant women on intermittent preventive treatment with sulfadoxine-pyrimethamine (IPTp-Sp) in Osogbo, southwest Nigeria. Blood sample was obtained from consenting pregnant women attending antenatal clinics. Microscopy and Polymerase chain reaction (PCR) were employed to diagnose and analyse genetic diversity. Of the 301 samples, 53 (18%) and 83 (28%) were positive for *P. falciparum* by microscopy and PCR, respectively. Using the merozoite surface protein (*msp)-1, msp-2*, and glutamate-rich protein (glurp) genes of *P. falciparum* as polymorphic markers, the msp-1 gene showed nine alleles with R033 (66.7%) being predominant, followed by K1 (45.5%) and MAD20 (33.3%). The msp-2 gene had 16 alleles (eight each for FC27 and 3D7). The 3D7 alleles (82.1%) was significantly more than FC27 alleles (48.2%) (p = 0.0093). Nine alleles were detected with glurp gene, presenting with the highest monoclonal and the lowest polyclonal infection. The multiplicity of infection (MOI) of 1.5, 1.8, and 1.2 were obtained for *msp-1*, *msp-2* and *glurp* genes. In light of the high *P. falciparum* genetic diversity among pregnant women on IPT-Sp in this study, additional strategies for preventing and controlling malaria in pregnancy might be required.

## Introduction

*Plasmodium falciparum* is the dominant *Plasmodium species* and the leading cause of malaria in sub-Saharan Africa^1^. Pregnant women form a subset of individuals highly susceptible to infection in endemic areas due to changes in acquired immunity and physiology associated with pregnancy^2^. Malaria in pregnancy has adverse consequences on the mother, the fetus, and the newborn^3^. In 2019, over 11 million pregnant women in sub-Saharan Africa were at risk of malaria, delivering about 822000 low birth weight children^1^. If undetected, it is capable of causing maternal anemia, retardation in fetal growth, preterm delivery, and low birth weight^3^,^4^. Currently, intermittent preventive treatment of malaria with sulfadoxine-pyrimethamine (IPTp-SP) and the use of long-lasting insecticide-treated bed nets (LLIN) are the mainstay for malaria control among pregnant women^5^. The World Health Organisation (WHO) recommends IPTp-SP for pregnant women at every antenatal (ANC) contact starting from 13 to 16 weeks, regardless of whether they have malaria infection or not, with each dose given at least four weeks apart^6^.

*Plasmodium falciparum* has a complex genetic structure that undergoes adaptation and conformational changes in the face of drug pressure, environmental changes, and host immune responses. This genetic diversity influences antimalarial drugs' transmission intensity, pathogenesis, and treatment outcome^7^. In high malaria-endemic settings, the multiplicity of infection (MOI), defined as the mean number of different *P. falciparum* strains infecting an individual, is an appropriate malaria metric determined by genetic diversity to provide useful information about malaria transmission in an area. The molecular surveillance of *P. falciparum's* genetic profile has become the focus of biomedical research as control measures are more selectively targeted towards the genetic makeup of the parasite^8^. The merozoite surface proteins (msp), specifically *msp-1* and *msp-2*, and glutamate-rich protein (*glurp*) are widely utilized biomarkers to elucidate the genetic structure of the parasite population in different settings. It is also used as a discriminatory tool to distinguish between recrudescent and new infections in drug efficacy trials^9^–^11^. The *msp-1, msp-2* and *glurp* genes are implicated in erythrocyte invasion and target immune response, making them candidates for blood-stage vaccine development^12^,^13^ and identifying genetically distinct multiple parasite clones within parasite sub-populations^14^,^15^. Extensive *P. falciparum* genetic diversity has been reported in different regions and patient categories^16^,^17^, with the findings revealing challenges to developing appropriate curative and preventive malaria interventions.

Despite the endemicity of malaria and its significant toll on pregnant women, there is inadequate data on *P. falciparum* genetic diversity among pregnant women in Nigeria. This study examined the genetic diversity, allelic frequency, and MOI of *P. falciparum* in pregnant women attending antenatal clinics in Osogbo, Nigeria, utilizing the *msp-1, msp-2* and *glurp* antigenic loci.

## Methods

### Study location and participants

**Figure 1:**
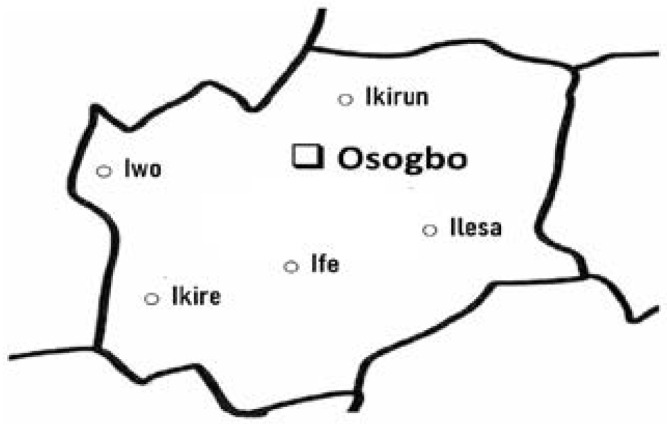
Map of Osun State showing Osogbo and some border communities in the state.

This cross-sectional study of pregnant women attending antenatal clinics in selected primary healthcare facilities was conducted in Osogbo, Osun-State in Southwestern Nigeria, from September 2015 to October 2016. Osogbo is the state capital of Osun State, and it represents a typical urban setting in Nigeria. Malaria transmission is seasonal, peaking during the rainy season from April to October. Osogbo shares boundaries with Ede, Ilesa and Obokun and is easily accessible from most of the state because of its central nature (Figure 1). The city was estimated to be 527,954 in 2017 at the official rate of 3% annual growth rate (National Bureau of Statistics, 2016). Inclusion criteria included pregnant women without fever in the last three days, no history of antimalarial treatment in the previous 14 days outside sulfadoxine-pyrimethamine, an axillary temperature of less than 37.5°C. According to National Malaria Control Program recommendations, all recruited pregnant women had taken second or third doses of IpT-Sp. A total of 301 asymptomatic pregnant women comprising 97 primigravidae and 204 multigravidae (age range 17-41 years), who gave informed consent were recruited into the study.

### Sample collection and processing

About 2 ml of venous blood were collected for Giemsa-stained thick and thin blood film and spotted on filter paper for PCR analysis. Two glass slides for thick and thin film microscopy were labeled for each participant and allowed to dry before staining with 5% Giemsa for 30 minutes, examined microscopically for the presence of malaria parasite. Parasites were counted against 200 white blood cells (WBCs) from the thick film. As an indicator of anemia, packed cell volume (PCV) was estimated by centrifuging the blood collected into a heparinised capillary tube. Anemia in pregnancy was defined as a haemoglobin level of less than 11 g/L (haematocrit ≤33%)^18^.

### Genomic DNA Extraction and PCR analysis

Genomic DNA was extracted from whole blood using the QIAamp DNA mini kit (Qiagen, Hilden, Germany), following the manufacturer's instructions. A previously described nested PCR protocol^19^ was utilized for the detection of *P. falciparum*. Briefly, the Plasmodium genus was amplified using the 18S ribosomal RNA in the first reaction, and the secondary reaction amplified the falciparum species using specific primers. A template-free control and a known positive sample were used as negative and positive controls, respectively.

### Allelic genotyping of Pf msp-1, msp-2 and glurp genes

*Plasmodium falciparum msp-1, msp-2*, and *glurp* genes were amplified using nested specific primers as previously described^20^. The primary reaction primers targeted the conserved regions of block 2, block 3, and R2 regions of *msp-1, msp-2* and *glurp* genes, while the nested reaction primers targeted the allelic families of *msp-1* (K1, MAD20, and R033), *msp-2* (3D7 and FC27) and *glurp* (R2) region, respectively. The PCR amplification for each allelic family for the primary and nested reactions were performed separately. Amplified PCR products were analysed by gel electrophoresis on a 1.5% agarose stained with SYBR Green and visualized by UV trans-illuminator (UVP® DigiDoc-It™, USA). Amplicon sizes were detected relative to the 100 bp molecular DNA ladder (New England Biolab).

### Multiplicity of infection

The MOI, defined as the average number of genotypes per infected individual, was calculated by dividing the total number of distinct *msp-1* and *msp-2* genotypes by the number of positive samples for each marker. Isolates with more than one allelic family were considered a multiclonal infection, while the presence of a single allelic family was considered a monoclonal infection.

### Statistical analyses

Data were analysed using SPSS software version 20. The allelic frequencies of *msp-1, msp-2* and *glurp* genes were calculated as the proportion of the allele detected for each allelic family out of the total alleles detected. The calculation of the frequency of multiclonal infection considered the number of samples harbouring more than one amplified clone out of the total sample. The proportionality assessment was performed using the Chi-square test, while Student's t-test was used to compare the MOI between allelic families. The level of statistical significance was considered at p < 0.05.

### Ethics

The study was conducted according to the standards of the 1964 Helsinki declaration. Ethical approval (approval number OSHREC/PRS/569^T^/33) was received from the Osun State Research and Ethics Committee, State Ministry of Health Osogbo, Nigeria. At the same time, written informed consent was obtained from pregnant women before enrolment into the study.

## Results

### Baseline characteristics

The study recruited 301 pregnant women who gave informed consent and were on intermittent preventive treatment with sulfadoxine-pyrimethamine for malaria prophylaxis. The mean age of the women was 27.2 ±5.04 years, while the mean PCV, indicative of anemia, was 33.1 ± 5.37%. 32.2% of the women were primigravidae and 67.8% multigravida (Table 1). Of the 301 blood samples collected, microscopy detected *P. falciparum* parasite positivity in 53 (17.6%), with a geometric mean parasite density of 917.53 parasites/µl of blood (range 120 - 9520). In addition, 83 (27.6%) samples were PCR positive.

**Table 1:** General characteristics of the study population

Characteristics	
Mean age ± SD	27.2 (range 17–41) ± 5.04 years
Mean PCV ± SD	33.1 ± 5.37 %
Gravidity	
Primigravidae	97 (32.2%)
Multigravidae	204 (67.8%)
Number positive by microscopy	53/301 (17.6%)
Geometric mean parasite density	917.53 (range 120 -9520)
Number positive by PCR	83/301 (27.6%)

SD= Standard deviation.

### Factors associated with malaria infection during pregnancy

The age group 17-24 years had the highest confirmed *P. falciparum* positivity rate (36.6%), followed by participants older than 35 years (31.0%), with the lowest (24.4%) among the age group, 25-34 years (Table 2). The difference in *P. falciparum* positivity among the age groups was not statistically significant (p = 0.33). Of the 83 samples positive by PCR for *P. falciparum*, 26 (26.8%) and 57 (27.9%) were primigravidae and multigravidae, respectively (p = 0.89). Anemia was present in 17 (32.1%) of the *P. falciparum*-positive women, while 66 (26.6%) of those positive did not have anemia (p = 0.49). On the use of insecticide-treated mosquito nets, 44 (25.9%) of women who reportedly slept under insecticide-treated bed nets were *P. falciparum* positive, while the remaining 126 (74.1%) were negative. Similarly, 39 (29.8%) of those who did not sleep under bed nets were also positive for *P. falciparum* (Table 2).

**Table 2:** Association between age, gravidity, anemia, and mosquito nets with respect to *Pf* positivity by PCR in the study population

Variables	*Plasmodium falciparum*			

	Positive (%)	Negative (%)	χ^2^	*p*-value
Age group				
17–24	30 (32.6)	62 (64.7)	2.22	0.33
25–34	44 (24.4)	136 (75.6)		
>35	9 (31.0)	20 (69.0)		
Gravidity				
Primigravidae	26 (26.8)	71 (73.2)	0.043	0.89
Multigravidae	57 (27.9)	147 (72.1)		
Anemia				
Yes	17 (32.1)	36 (67.9)		
No	66 (26.6)	182 (73.4)	0.65	0.49
Mosquito net				
Yes	44 (25.9)	126 (74.1)	0.560	0.52
No	39 (29.8)	92 (70.2)		

### Allelic frequency distribution of *msp-1*, *msp-2* and *glurp* genes

The *msp-1* gene had nine distinct allelic types, with R033 being the most predominant allelic family (66.7%), followed by K1 (45.5%) and MAD20 (33.3%). Fragment sizes ranged from 200 – 600 bp for K1-type, 200 – 300 bp for MAD20-type, and 150 – 200 bp for R033-type. Combined allelic frequency was more with K1 + R033 combination (21.2%) compared to other *msp-1* allelic combinations (p = 0.002). For *msp-2* gene, 16 alleles were detected, with 8 alleles each for FC27 and 3D7 families. Fragment size for FC27 ranged from 300 – 900 bp, while 3D7 was 200 – 900 bp. The predominant allelic frequency occurred in 3D7 (82.1%), with FC27 recording the least frequency (48.2%). There was a significant difference in the number of amplified alleles between FC27 and 3D7 (p = 0.009). Of the thirty-five isolates positive for the *glurp* gene, nine distinct alleles were identified, with fragment sizes ranging from 300 – 1100 base pairs. Overall, *msp-2* was the predominant gene, while *glurp* had the least allelic frequency distribution (Table 3).

**Table 3:** Genetic diversity of *Plasmodium falciparummsp*-1, *msp*-2 and *glurp* genes

Gene	Allele	Baseline			
		Positive	*p*-value	Fragment	No. of
		(n/N) (%)		size (bp)	alleles
*msp*-1	K1	15/33 (45.5)		200–600	5
(33/83)					
	MAD20	11/33 (33.3)		200–300	3
	R033	22/33 (66.7)		150–200	2
	K1+MAD20	3/33 (9.1)	0.0019*		
	K1+R033	7/33 (21.2)			
	MAD20+R033	6/33 (18.2)			
	K1+MAD20+R033	2/33 (6.1)			
*msp*-2	FC27	27/56 (48.2)		300–900	8
(56/83)			0.0093*		
	3D7	46/56 (82.1)		200–900	8
	FC27+3D7	18/56 (32.1)			
*glurp* (35/83)		35/35 (100)		300–1100	9

### Multiplicity of infection

Among the *P. falciparum* isolates, monoclonal infections were commoner than polyclonal infections. The highest monoclonal infection occurred in *glurp* (77.1%) with 1.2 MOI. The *msp-2* gene had the highest polyclonal infection of 44.6% among the three genes and 55.4% monoclonal infection, with an MOI of 1.8. Similarly, *msp-1* recorded 60.6% monoclonal infections and 39.4% polyclonal infections with MOI of 1.5. There was no significant difference in the three genes between the mono- and polyclonal infections (Table 4).

**Table 4:** Multiplicity of infection of *P. falciparum msp*-1, *msp*-2 and *glurp* genes among pregnant women

	MOI	Monoclonal infection % (n/N)	Polyclonal infection % (n/N)
*msp*-1	1.5	60.6 (20/33)	39.4 (13/33)
*msp*-2	1.8	55.4 (31/56)	44.6 (25/56)
*glurp*	1.2	77.1 (27/35)	22.9 (8/35)
p-value		0.099	0.092

### Comparison of age with allelic frequencies in *msp-1, msp-2* and *glurp* genes

Comparing different age groups with the number of alleles detected revealed that R033 and MAD20 were predominant in the age group 17 – 24 years, while 3D7, FC27 and *glurp* R2 region dominate the age group 25 – 32 years. The lowest allelic frequency was detected in the age group 33 – 41 years, with no significant difference in the MOI among the three age groups.

## Discussion

This study reported the prevalence, genetic diversity, and MOI of *P. falciparum* isolates from asymptomatic pregnant women on IPT-Sp attending antenatal clinics in Osogbo, Southwest Nigeria. Using the PCR technique, 28% of the pregnant women who reportedly used SP were positive for *P. falciparum*, raising concern about SP's efficacy for malaria prevention in pregnancy. This observation corroborated our recent study that reported a high prevalence of SP-resistant markers among pregnant women in a different part of Southwest Nigeria, emphasizing the need to start considering alternative IPT strategies^21^.

The highest *P. falciparum* positivity rate occurred in the younger age group (17 – 24 years) followed by > 35 years and 25 – 34 years, although the differences were not statistically significant. Published reports from other African countries have consistently shown that the most significant risk of malaria infection and highest parasite densities among pregnant women were recorded among younger age groups^22^,^23^. Older age groups seem to have had improved exposure to health services and better awareness about malaria infection and prevention. Besides, previous malaria exposures in older mothers inarguably might have endowed a better-acquired immunity to malaria^23^. Despite these factors, other studies have reported no significant association between age and malaria^24^,^25^, implying that other factors outside immunity and awareness might be responsible.

A comparison of malaria infection with gravidity (primigravidae versus multigravidae) did not show any significant association in this study, agreeing with a previous study^26^. On the contrary, other studies have presented strong evidence that multigravid women are less prone to malaria, while disease severity is likely more associated with primigravidae^22^,^27^. Immunological factors depicting the benefits of previous malaria exposures or pregnancy( ies) are also associated with gravidity^2^,^28^.

The *P. falciparum* genetic diversity is essential for the malaria vaccine development process and provides epidemiological information on transmission intensity in a given region. The genotyping of the three distinct genes encoding *msp-1, msp-2* and *glurp* revealed a high degree of genetic diversity, with *msp-2* recording the highest MOI (1.8). The observed high genetic diversity agrees with high malaria transmission in Nigeria and most of sub-Saharan Africa. In comparison, this study's relatively high MOI is consistent with a recent report that attributed this to the widespread use of insecticide-treated bed nets 21 and IPT-Sp, leading to reduced exposure of pregnant women to malaria. As observed in this study, all pregnant women are on IPT-Sp, and a high percentage reportedly used insecticide-treated bed nets. However, a relatively high rate (26%) of these women also recorded positive malaria parasitemia. Since the response to the use of insecticide bed nets was not verified in the participants' homes, this observation has to be interpreted with caution as previous studies have reported low insecticide bed nets^29^ and LLIN^30^,^31^ use among pregnant women in Nigeria.

The R033 family was the most predominant allele of the *msp-1* gene in the study. This observation was similar to our recent report in Ibadan, a neighbouring city to Osogbo 32. However, reports from other parts of southwest Nigeria have reported the K1 family as the most predominant *msp-1* allele^33^,^34^. With the K1 family being the second most predominant allelic family in our study, we can conclude that the two families are the dominant alleles in southwest Nigeria. Although this study did not investigate clinical symptoms since the pregnant women were asymptomatic, previous studies have associated a high frequency of R033 alleles with asymptomatic and mild malaria^35^,^36^. In contrast, others have not established the correlation of *msp-1* allelic families to clinical forms of malaria^37^,^38^.

For the *msp-2* gene, the FC27 allelic family was the most commonly observed allele, contrary to our previous report showing 3D7 as the most common *msp-2* allele among children with uncomplicated malaria in this area^39^. Another of our studies in Ibadan among children with uncomplicated malaria, on the other hand, agreed with our current analysis with FC27 as the most prevalent allele^32^. Like *msp-1*, the reported trends of the differential distribution of 3D7 and FC27 alleles concerning clinical status have yielded conflicting results making it difficult to establish the actual influence of the *msp-2* gene allelic families on clinical course. The 3D7 allele is assumed to protect against the clinical disease since it is frequently encountered in asymptomatic malaria^35^,^40^, an observation that holds forth in this study but contrasted by others^10^,^41^, also considering that the women were followed up for two weeks after recruitment and they remained asymptomatic.

The *glurp* gene recorded the highest monoclonal and the lowest polyclonal parasite infection with nine distinct alleles and the least MOI among the three genes studied. Our previous report among children with uncomplicated malaria in this same location^42^ and within this region^32^ revealed more distinct alleles. The lower number of alleles and low MOI might be due to the different interventions (insecticide nets and IPT-Sp) among these pregnant women, resulting in the reduction of parasite transmission intensity and genetic diversity. Although this seems to be an encouraging observation, the fact that these parasites are still circulating in the face of these deployed interventions calls for worries and the need to initiate early strategies for alternative malaria control approaches.

A limitation of our study is the inability to supervise SP intake by pregnant women directly. The women were given the drugs freely to take home in some cases, and they confirm the use of the drug in the administered questionnaire, but their compliance cannot be independently verified.

## Conclusion

Genetically diverse *Plasmodium falciparum* strains with different clones are infecting pregnant women without clinical symptoms in Nigeria. A high prevalence of polyclonal infections was observed for the three markers, with *msp-2* allelic families showing the highest polyclonality and the highest genetic diversity. The high prevalence of asymptomatic *P. falciparum* and the genetic diversity in this study indicate sustained transmission. This study draws attention to the need to intensify efforts in identifying newer chemoprophylactic methods for malaria in pregnant women in Nigeria in addition to the existing ones. Also, rewards may be introduced to facilitate compliance with the use of SP for the intermittent preventive treatment of malaria in pregnancy.
